# A designable twist-densification route to bioactive collagen hydrogel yarns approaching tendon-like mechanics

**DOI:** 10.1016/j.bioactmat.2026.09.010

**Published:** 2026-09-17

**Authors:** Yang Xie, Wenjie Wu, Weiwei Zhang, Xiangjun Peng, Tiancai Sun, Yanling Liu, Zuoqi Zhang, Elliot L. Elson, Guy M. Genin, Guoyou Huang

**Affiliations:** a Department of Engineering Mechanics, School of Civil Engineering, Wuhan University, Wuhan, China; b School of Mathematics and Physics, China University of Geosciences, Wuhan, China; c Department of Engineering Mechanics, Applied Mechanics Laboratory, Institute of Biomechanics and Medical Engineering, Tsinghua University, Beijing, China; d College of Chemistry and Molecular Sciences, Wuhan University, Wuhan, China; e NSF Science and Technology Center for Engineering Mechanobiology, Department of Mechanical Engineering and Materials Science, Washington University, St. Louis, MO, USA

**Keywords:** Collagen hydrogels, Superhelical architecture, Twist-induced densification, Textile tissue engineering, Mechanical enhancement

## Abstract

Reconstituted collagen hydrogels offer the bioactivity that load-bearing tissue engineering requires, but their Pascal to low-kPa moduli have confined them to non-structural roles, and the synthetic and hybrid strategies that close the mechanical gap typically forfeit that bioactivity. We show that a twist-induced densification process resolves this tradeoff in a controllable way, converting soft collagen hydrogel fibers into superhelical hydrogel yarns whose mechanics can be prescribed from fabrication parameters. A parameter-free model drawn from fiber-network mechanics predicts the modulus enhancement from densification, fibril alignment, and helical fiber architecture, and the same surface helix angle independently predicts the nonlinear strain-stiffening response. The resulting yarns exhibit modulus, strength, and toughness approaching the lower range reported for native tendons, representing enhancements of two to three orders of magnitude over the as-fabricated collagen hydrogels. Importantly, they remain amenable to braiding, knitting, and weaving into two- and three-dimensional constructs, including tubular architectures that recover elastically under repeated compression. Short-term cytocompatibility is preserved despite the severe compaction: encapsulated fibroblasts retain over 90% viability, exhibit pronounced alignment within the yarns, and transduce externally applied strain. Twist densification thus provides a designable route to living, load-bearing protein textiles.

## Highlights

• Superhelical collagen hydrogel yarns approaching tendon-like mechanics are developed.
• A parameter-free model drawn from fiber-network mechanics predicts the modulus enhancement.
• The hydrogel yarns enable textile processing into varied architectures.
• The hydrogel yarns support three-dimensional cell culture and mechanical cue transmission.

## Graphical abstract

**Figure:**
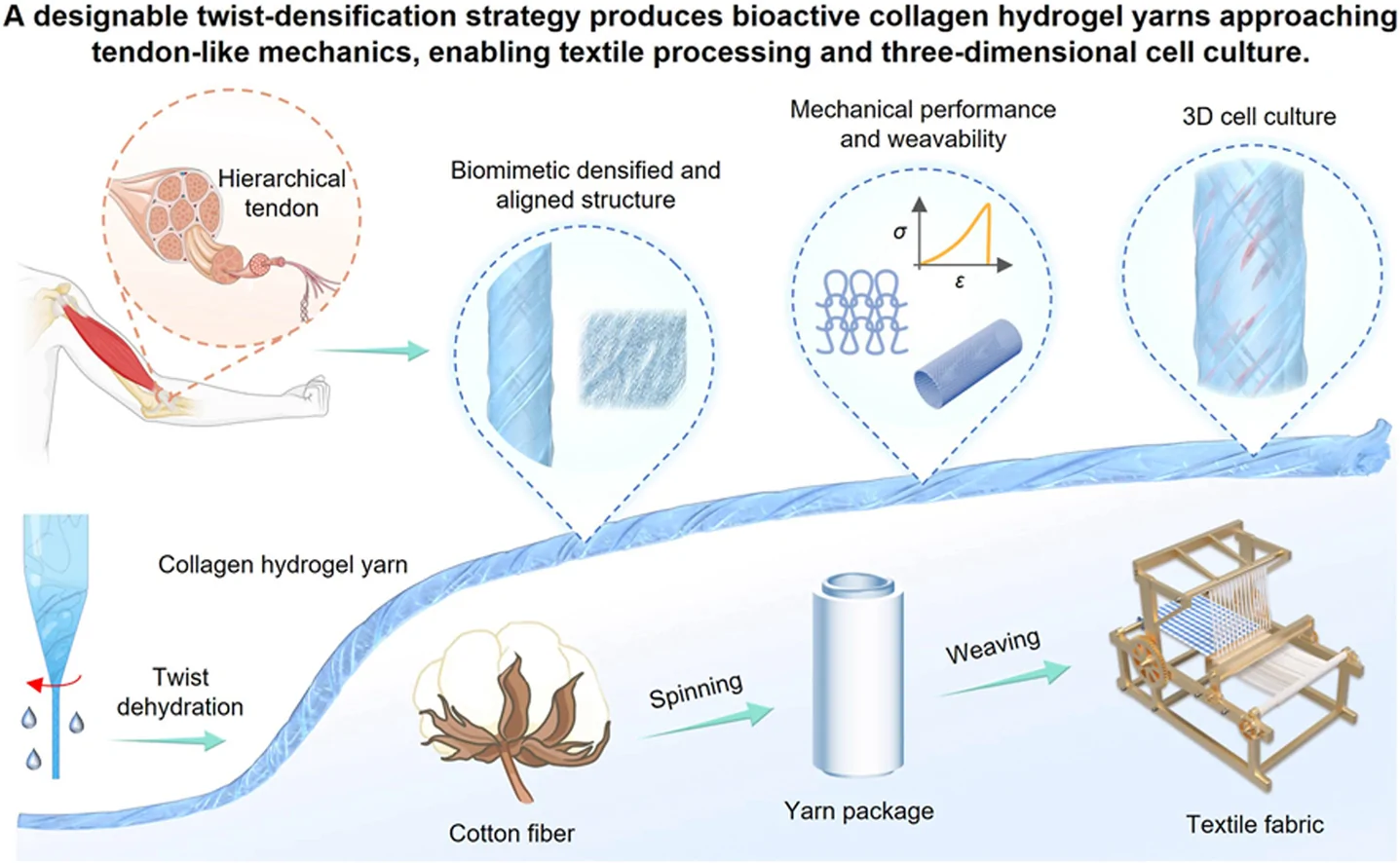

## 1 Introduction

Load-bearing soft tissues such as tendons and ligaments derive their mechanical performance from hierarchical fibrous architectures spanning molecular to macroscopic length scales, in which collagen, the principal structural protein in the body, is organized into aligned, densely packed fiber bundles [[1], [2], [3], [4], [5], [6]]. Reconstituted collagen hydrogels can recapitulate much of the native biochemical microenvironment, supporting cell adhesion, proliferation, and matrix remodeling, and have consequently become central to tissue engineering and regenerative medicine [[7], [8], [9]]. However, a fundamental tradeoff between mechanical and biological performance has constrained their practical application: at the concentrations compatible with cell encapsulation (typically 1-6 mg/mL), collagen hydrogels exhibit tensile moduli in the low-kPa range, three to four orders of magnitude below the MPa-scale stiffness of native tendon [[10], [11], [12], [13], [14], [15]]. Bio-inspired strategies employing synthetic or hybrid hydrogels have achieved impressive mechanical properties, but typically at the cost of bioactivity, requiring either bioinert polymers or processing conditions incompatible with cell viability [[16], [17], [18], [19], [20], [21], [22], [23]].

Several strategies have been pursued to narrow this mechanical gap while preserving the intrinsic bioactivity of collagen. Structural densification through plastic compression, mechanical loading, or chemical crosslinking can compact collagen networks and promote fiber alignment, yielding constructs with substantially enhanced stiffness and toughness that remain compatible with three-dimensional (3D) cell culture [[24], [25], [26], [27]]. Our previous study further demonstrated that twisting and crosslinking can substantially strengthen collagen hydrogels and enable the fabrication of simple tissue constructs [28]. However, their implementation has so far been largely limited to relatively simple geometries, with limited control over tissue-scale architecture, while the mechanisms underlying mechanical reinforcement remained insufficiently resolved. Bioprinting technologies provide complementary architectural versatility but have struggled to achieve the mechanical thresholds required for load-bearing applications, owing to low solid content and weak interfilament bonding in printable collagen formulations [[29], [30], [31], [32]]. Traditional textile fabrication offers a compelling alternative by providing fiber interlocking, tunable anisotropy, and hierarchical load distribution [[33], [34], [35], [36], [37], [38]]. However, textile approaches to tissue engineering have been constrained by the lack of hydrogel yarns that combine the tensile strength needed to survive textile processing with the bioactivity needed to support encapsulated cells [[39], [40], [41], [42]].

**Fig. 1:**
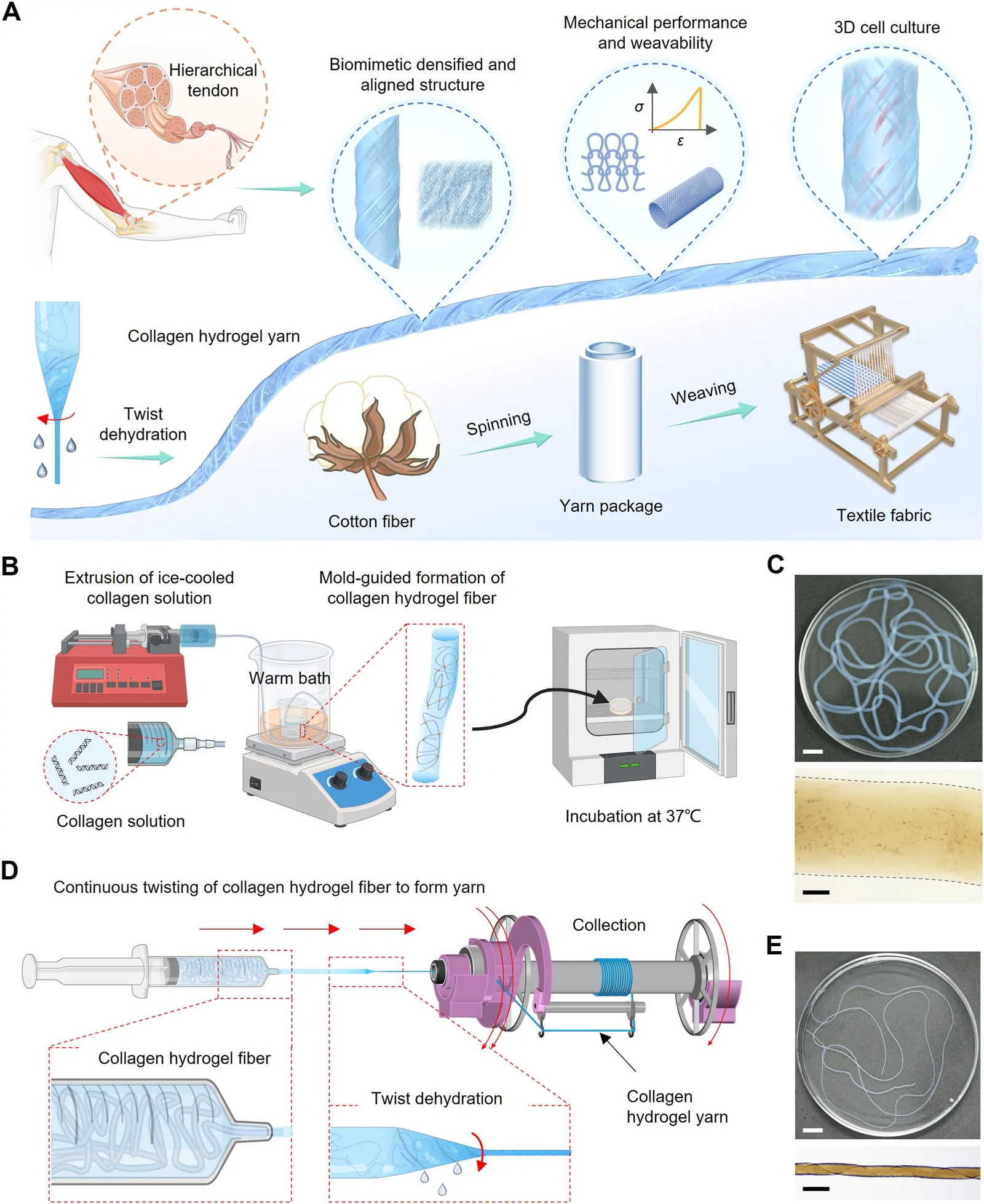
Twist-induced densification transforms dilute collagen hydrogel fibers into superhelical yarns with tissue-scale density. (**A**) Schematic overview of the fabrication strategy and its structural analogy to native tendon hierarchy. Collagen hydrogel fibers (ColHFs; diameter ∼1800 μm, solid fraction $\varphi_{0}\approx$ 0.003) are twisted into collagen hydrogel yarns (ColHYs; diameter ∼200 μm, φ≈ 0.2–0.3), achieving a ∼9-fold reduction in radius and ∼80-fold increase in solid fraction. (**B**) Fabrication of ColHFs by thermal self-assembly of neutralized collagen solution (4 mg/mL) within silicone tube templates at 37°C. (**C**) Optical images of ColHFs. Scale bars: 1 cm (upper), 500 μm (lower). (**D**) Twist-induced densification of pre-crosslinked ColHFs using a miniature spinning apparatus. Continuous torsional strain drives radial compaction and water expulsion, transforming the dilute hydrogel into a dense, superhelical yarn. (**E**) Optical images of the resulting ColHYs. Scale bars: 1 cm (upper), 500 μm (lower). The tendon anatomy panel in (A) was obtained from Servier Medical Art (CC BY 4.0). Parts of this figure were created using BioRender.com.

Here, we demonstrate that a simple twist-induced densification process transforms soft collagen hydrogel fibers (ColHFs) into high-performance yarns with a hierarchical superhelical architecture that recapitulates the structural motifs of native tendon (Fig. 1A). The resulting collagen hydrogel yarns (ColHYs) exhibit tensile moduli, strengths, and toughness values enhanced by two to three orders of magnitude relative to the as-fabricated collagen hydrogels, while preserving cell-supportive properties and efficient matrix-to-cell strain transfer. The dramatic enhancements are predictable. By comparing intermediate processing states that isolate geometric transformation from chemical crosslinking, we demonstrate that three factors dominate: network densification (set by the radial compaction ratio), fibril alignment (the random-to-oriented transition), and the helical fiber architecture inherent to twisting [43]. The surface helix angle that enters the stiffness prediction independently predicts nonlinear strain-stiffening analogous to that which characterizes tendon [44,45]. This quantitative framework renders twist-induced densification a designable process, in which mechanical properties can be rationally predicted and tuned from process parameters. It also establishes twist densification as a general strategy for overcoming the mechanics-bioactivity tradeoff in reconstituted protein hydrogels. We further demonstrate that ColHYs possess the textile-grade mechanical performance required for weaving, knitting, and braiding into 2D and 3D architectures, and that encapsulated cells within these yarns exhibit high viability, contact-guided alignment, and strain-responsive morphological adaptation.

## 2 Materials and methods

### 2.1 Materials

Ethyl alcohol, K_2_PtCl_4_, and genipin were obtained from Macklin (China). Cell culture reagents, including Dulbecco's Modified Eagle's Medium (DMEM), fetal bovine serum (FBS), penicillin/streptomycin (PS), and trypsin, were supplied by Gibco (USA). For cell viability assays, the Calcein-AM/PI Double Staining Kit was purchased from Dojindo (Japan). Fixation and staining reagents, including 4% paraformaldehyde, Triton X-100, rhodamine phalloidin, and 4′,6-diamidino-2-phenylindole (DAPI) solution, were sourced from Solarbio (China). Type I collagen was either extracted from rat tails obtained from Powerful Biology (China) or kindly supplied by Guangzhou Trauer Biotechnology (acid-soluble bovine tendon collagen solution; China). Phosphate-buffered saline (PBS) was prepared by dissolving PBS tablets (Solarbio, China) in deionized water. NIH/3T3 fibroblasts were obtained from the Cell Bank of the Chinese Academy of Sciences (China). Sprague-Dawley rat bone marrow-derived mesenchymal stem cells (rBMSCs; catalog no. STCC5011; Wuhan Zishan Biotechnology Co., Ltd., China) were used between passages 3–6. Deionized water was used throughout the experiments.

### 2.2 Fabrication of ColHFs and ColHYs

To prepare ColHFs, a neutralized collagen solution (4 mg/mL) was prepared at 4°C. For each milliliter of the solution, 666.7 μL collagen (6 mg/mL) was blended with 100 μL of 10× PBS, 76 μL of 0.2 mol/L NaOH, and 157.2 μL of sterile deionized water. This mixture was loaded into a syringe and maintained at low temperature using ice cooling to prevent premature gelation. The collagen solution was subsequently extruded via an infusion pump (LD-P2020, LANDEYILIAO, China) at a controlled flow rate of 25 mL/h. The syringe outlet was interfaced with silicone tubing, the terminal end of which was submerged in PBS buffer preheated to 37°C. Upon extrusion into the heated environment, the neutralized collagen underwent rapid thermally induced self-assembly. Following initial gelation within the tubing, the constructs were transferred to a 37°C incubator (SN-SPX-30B, SUNNE, China) for further incubation, ensuring the formation of mature, fully self-assembled ColHFs.

The as-prepared ColHFs were initially submerged in a 0.1 mmol/L K_2_PtCl_4_ or 0.1 mmol/L genipin solution at 37°C for 10 min to achieve partial crosslinking, yielding pre-crosslinked ColHFs (pColHFs). This step ensured sufficient structural integrity for subsequent mechanical manipulation. The pColHFs were then collected and anchored at one end, with the other end secured to the spindle of a modified spinning apparatus (Feishi, China). Continuous torsional strain caused the fibers to twist and wrap around the spindle (Movie S1), expelling water and compacting the collagen network to form nascent yarns (nColHYs). To stabilize the remodeled architecture, the nColHYs were subjected to a second crosslinking step, either in 0.1 mmol/L K_2_PtCl_4_ at 37°C for 1 h or in 0.1 mmol/L genipin at 37°C for 24 h. The resulting ColHYs were washed and stored in PBS for further characterization.

To fabricate helical ColHYs, the prepared nColHYs were tightly wrapped around a stainless-steel acupuncture needle, serving as a cylindrical template. Both ends were carefully secured, and continuous hydration was maintained throughout the manipulation process to preserve the hydrogel's structural integrity. Following assembly, the template-bound samples were submerged in a 0.1 mmol/L K_2_PtCl_4_ solution for an additional hour of crosslinking at 37°C, ultimately yielding helical ColHYs after removal from the template.

### 2.3 Textile fabrication

All textiles were handcrafted using braiding, knitting, or weaving techniques. Braided structures were produced by interlacing eight yarns at a controlled braiding angle. Funnel and tubular structures were produced using weaving, in which the weft and warp yarns were interlaced at a 90° angle. Specifically, the tubular structures consisted of 16 warp yarns wrapped along the length of a stainless-steel column, with a single weft yarn woven circumferentially across the warp yarns, forming an almost seamless tubular fabric. For the braided tendon-like, funnel, and tubular structures, a custom-built polytetrafluoroethylene frame was employed to maintain structural integrity and provide mechanical support. Hydration was consistently maintained throughout the textile fabrication process.

### 2.4 Structural characterization

For microstructure examination, hydrogel specimens were first fixed in 4% paraformaldehyde for 1 h, followed by a 6 h rinse in PBS. To preserve fibrillar architecture, the samples underwent sequential dehydration in a graded ethanol series (30%, 50%, 70%, 90%, and 100% v/v) for 15 min each. This was followed by a solvent exchange process using graded tert-butanol solutions (30%, 50%, 70%, 90%, and 100% v/v). The samples were subsequently lyophilized in a freeze-dryer (SCIENTZ-10N, China) for complete solvent removal. The morphology was characterized using a scanning electron microscope (SEM) (Tescan MIRA, Tescan, Czech Republic) at an accelerating voltage of 5 keV. Prior to imaging, all samples were sputter-coated with gold. Micrographs were captured at varying magnifications (50×, 200×, 5000×, and 10000×) to analyze the hierarchical structure. ImageJ software was utilized to quantify fibril orientation. The direction of the macroscopic threads adjacent to the collagen fibrils being analyzed was defined as 0°, with clockwise deviations designated as positive angles. To further assess the structural anisotropy and long-range order of the collagen fibrils, ColHY specimens were equilibrated in PBS and observed under a polarized light microscope (Axio Observer 7m, Carl Zeiss, Germany) to evaluate their characteristic birefringence.

### 2.5 Mechanical characterization

All tensile properties were evaluated using a custom-built mechanical testing platform. Prior to characterization, the samples were equilibrated in 1× PBS to achieve swelling equilibrium, and their respective cross-sectional diameters were measured using an optical microscope (CKX53, Olympus, Japan). To simulate physiological conditions and prevent dehydration, all tests were performed in a PBS bath at a displacement rate of 25 μm/s, corresponding to a nominal engineering strain rate of approximately 0.3%/s under the testing geometry used in this study. Engineering stress was calculated using the initial cross-sectional area of each equilibrated specimen.

For the quasi-static tensile tests of individual yarns (Movie S2), the toe-region and linear-region moduli were determined from the slopes between the selected endpoints of the corresponding regions of the engineering stress–strain curve. Toughness was calculated as the area under the engineering stress–strain curve up to failure. Knot-retention tests were performed on knotted ColHYs using the same quasi-static tensile protocol. Knot-strength retention was calculated by normalizing the mean ultimate tensile strength of the knotted ColHYs to that of the unknotted ColHYs. For the stress-relaxation assays, specimens were subjected to a 20% tensile strain at a rate of 25 μm/s, and the resulting stress decay was monitored. In the constant-elongation elastic-recovery test, ColHYs were initially preloaded to 5 mN and then stretched to 20% strain. This strain was held for 12 min to observe stress dissipation, followed by unloading to the initial position. After a further 12 min recovery period, the samples were re-stretched until the tensile force exceeded the baseline, allowing for the deconvolution of elastic and plastic deformation components.

Cyclic tensile tests were performed on both nColHY and ColHY specimens at loading and unloading rates of 25 μm/s. For each group, the maximum load (*F*_max_) was set to 80% of the corresponding mean fracture load determined from quasi-static tensile tests, whereas the minimum load (*F*_min_) was set to 2% of the mean fracture load, giving a load ratio of *F*_min_/*F*_max_ = 0.025. A small non-zero *F*_min_ was applied to maintain the compliant yarns under slight tension throughout cycling and thereby prevent slackening or buckling during unloading, while minimizing pre-tension that could interfere with residual-strain measurements. At both the maximum and minimum load levels, the specimens were held for 150 s before the subsequent loading or unloading step. Residual strain was determined at a tensile force corresponding to 8% of the prescribed *F*_max_ during each loading phase. This protocol was designed to compare the relative cyclic mechanical stability of nColHYs and ColHYs under controlled loading conditions rather than to reproduce a specific physiological loading regime.

For long-term stability assessment, ColHYs were incubated in complete culture medium at 37°C and 5% CO_2_ for 28 days and mechanically evaluated at days 0, 14, and 28. Strength retention at each time point was calculated by normalizing the ultimate tensile strength to the mean day-0 ultimate tensile strength and was expressed as a percentage.

To enable reproducible gripping and quantitative mechanical characterization of the textile construct, a simplified knitted ColHY construct was fabricated and tested using the same mechanical testing platform and quasi-static displacement rate as those used for individual yarns. Before tensile testing, a preload of 5 mN was applied to remove slack from the knitted construct. The gauge length measured under this preload was defined as the initial gauge length. Because three yarns spanned the load-bearing cross-section of the construct, the effective initial cross-sectional area was defined as the sum of the initial cross-sectional areas of these three constituent yarns. Engineering stress was calculated by dividing the tensile force by this effective cross-sectional area.

For suture-pullout testing, a suture was passed through the simplified knitted construct, with one grip holding the suture and the opposing grip holding the construct. The specimen was pulled at the same quasi-static loading rate until suture pullout or construct failure, and the maximum pullout force was recorded. The suture-retention ratio was calculated as the mean maximum suture-pullout force divided by the mean maximum tensile failure load of the simplified knitted constructs. Cyclic tensile testing was performed using the same normalized loading protocol as that used for individual ColHYs.

### 2.6 Degradation analysis

To evaluate collagenase-mediated degradation, type I collagenase (Collagenase I; BioFroxx, China) was dissolved in complete culture medium at a final concentration of 0.5 mg/mL. nColHY and ColHY specimens were individually immersed in 1 mL of this solution in 24-well culture plates and incubated at 37°C and 5% CO_2_. To monitor dimensional changes during degradation, sample diameters were measured every 20 min using an optical microscope. Diameters at each time point were normalized to their respective initial diameters at 0 min.

### 2.7 Cell culture

DMEM was mixed with FBS at a volume ratio of 10:1 and supplemented with 1% (v/v) PS to prepare complete culture medium. The medium was refreshed every 2–3 days throughout the cell culture period. Cells were cultured in complete medium in a temperature-controlled CO_2_ incubator (Heal Force, China). When cell confluence reached approximately 85%, adherent cells were digested with trypsin and subsequently collected by centrifugation using a low-speed centrifuge (Hunan Pingke Scientific Instruments, China). The harvested cells were resuspended in fresh complete medium to obtain a cell concentration of 1 × 10^6^ cells/mL. This suspension was then thoroughly mixed with a neutralized collagen solution to yield a final mixture with a cell concentration of 2 × 10^5^ cells/mL and a collagen concentration of 4 mg/mL. The cell–collagen mixture was immediately loaded into a syringe and slowly extruded using an infusion pump. Following the previously described ColHF preparation protocol, the extruded mixture was incubated at 37°C for 45 min to allow gelation. Subsequently, cell-loaded ColHYs were fabricated according to the established ColHY preparation method. The resulting constructs were transferred to complete culture medium for further incubation. All tools and materials were sterilized before use.

### 2.8 Cell viability analysis

To evaluate the cytocompatibility of the ColHYs, a Calcein-AM/PI dual-staining assay was performed to distinguish between live and dead cells. Throughout the procedure, stringent light-shielding measures were implemented to prevent fluorophore photobleaching. Briefly, cell-laden specimens were harvested from the culture medium and gently rinsed twice with 1× PBS (5 min per wash) to remove residual serum components. The specimens were then incubated in a working solution containing 3 μM Calcein-AM and 4.5 μM PI for 15 min at 37°C. Following incubation, the stained constructs were visualized using a fluorescence microscope equipped with appropriate excitation and emission filters (blue for Calcein-AM, green for PI). The cell viability ratio, defined as the proportion of viable cells relative to the total cell population, was calculated and quantitatively analyzed using ImageJ software.

### 2.9 Cell spreading analysis

To evaluate cell morphology, cytoskeletal staining was performed on cell-loaded specimens. Briefly, samples were fixed with 4% paraformaldehyde at room temperature for 1 h, followed by three rinses with 1× PBS (10 min per rinse). The specimens were then permeabilized with 0.5% Triton X-100 for 15 min and rinsed twice with 1× PBS (10 min per rinse). Subsequently, the permeabilized samples were incubated with rhodamine phalloidin (200 nM) at room temperature for 45 min under dark conditions, followed by two rinses with 1× PBS (10 min per rinse). Nuclear staining was then performed by incubating the specimens with 10 μg/mL DAPI at room temperature for 30 min in the dark. After two additional rinses with 1× PBS (10 min per rinse), the samples were mounted onto glass slides using an anti-fade mounting medium and imaged using a confocal laser scanning microscope (NCF1000, NEXCOPE, China). Cell spreading area and aspect ratio were quantified using ImageJ. The control (Ctrl) condition consisted of cell-laden ColHFs immediately after cell encapsulation and before twist-induced densification.

For the stretching experiment of cell-laden ColHYs, samples cultured for 3 days were held at 0%, 7%, or 15% tensile strain for 1 h. The samples were then fixed and stained, and cell aspect ratio was quantified as described above.

### 2.10 Statistical analysis

Unless otherwise stated in the corresponding figure legend, quasi-static mechanical comparisons of ColHFs, pColHFs, nColHYs, and ColHYs were based on three independent fabrication batches prepared on separate occasions (n = 3). For each material state, five technical replicate specimens from each batch were mechanically tested, and their mean was treated as one independent data point. For cell-based experiments, five independent experiments were performed on separate occasions (n = 5), with two technical replicate specimens included in each experiment and two randomly selected non-overlapping fields of view acquired from each specimen. For live/dead analysis, at least 150 cells per specimen were included in the final count. For cytoskeletal and nuclear-staining analyses, only cells with clearly resolved boundaries and complete morphological information were included in the analysis. Individual-cell measurements were first averaged to obtain a specimen-level value, and the two specimen-level values were then averaged to yield one independent data point for each experiment. A two-tailed unpaired Student's t-test was used for comparisons between two groups, whereas Spearman's rank correlation analysis was used to assess the monotonic association between culture time and cell spreading area or aspect ratio from day 0 to day 7, with both analyses executed in Origin. Differences between groups were considered statistically significant at *p* < 0.05. All experimental data are presented as the mean ± standard deviation (SD).

## 3 Results and discussion

### 3.1 Superhelical architecture from twist-induced densification

Collagen hydrogel fibers (ColHFs) were prepared by thermal self-assembly of neutralized collagen solution (4 mg/mL) within silicone tube templates (Fig. 1B and C; see Materials and Methods for details). The resulting ColHFs were soft, hydrated fibers ∼1800 μm in diameter, and were lightly crosslinked with K_2_PtCl_4_ or genipin to provide sufficient handling strength [46], then subjected to continuous torsional strain using a miniature spinning apparatus (Fig. 1D and E; Movie S1). This twisting process simultaneously expelled water from the collagen network and compacted the fiber radially, producing collagen hydrogel yarns (ColHYs) with diameters of ∼200 μm or less, a ∼9-fold reduction in radius and ∼80-fold reduction in cross-sectional area. A secondary crosslinking step stabilized the remodeled architecture. The final yarn diameter could be controlled by varying the template tube diameter (Fig. S1). For clarity, intermediate states are denoted pColHFs (pre-crosslinked hydrogel fibers) and nColHYs (nascent hydrogel yarns before secondary crosslinking); unless otherwise stated, ColHYs with diameters of ∼200 μm were used throughout.

The structural consequences of this compaction were striking. At the initial concentration of 4 mg/mL (collagen density ≈ 1.35 g/cm^3^), the ColHF solid volume fraction was only $\varphi_{0}\approx$ 0.003, a sparse, mechanically fragile network. The 80-fold area reduction drove this to φ≈ 0.2-0.3, consistent with measured mass fractions (Fig. S2) and approaching the solid content of native tendon and ligament [47,48]. This transformation from a dilute hydrogel to a dense, tissue-scale fibrous material is the central physical process underlying the mechanical enhancement quantified below.

**Fig. 2:**
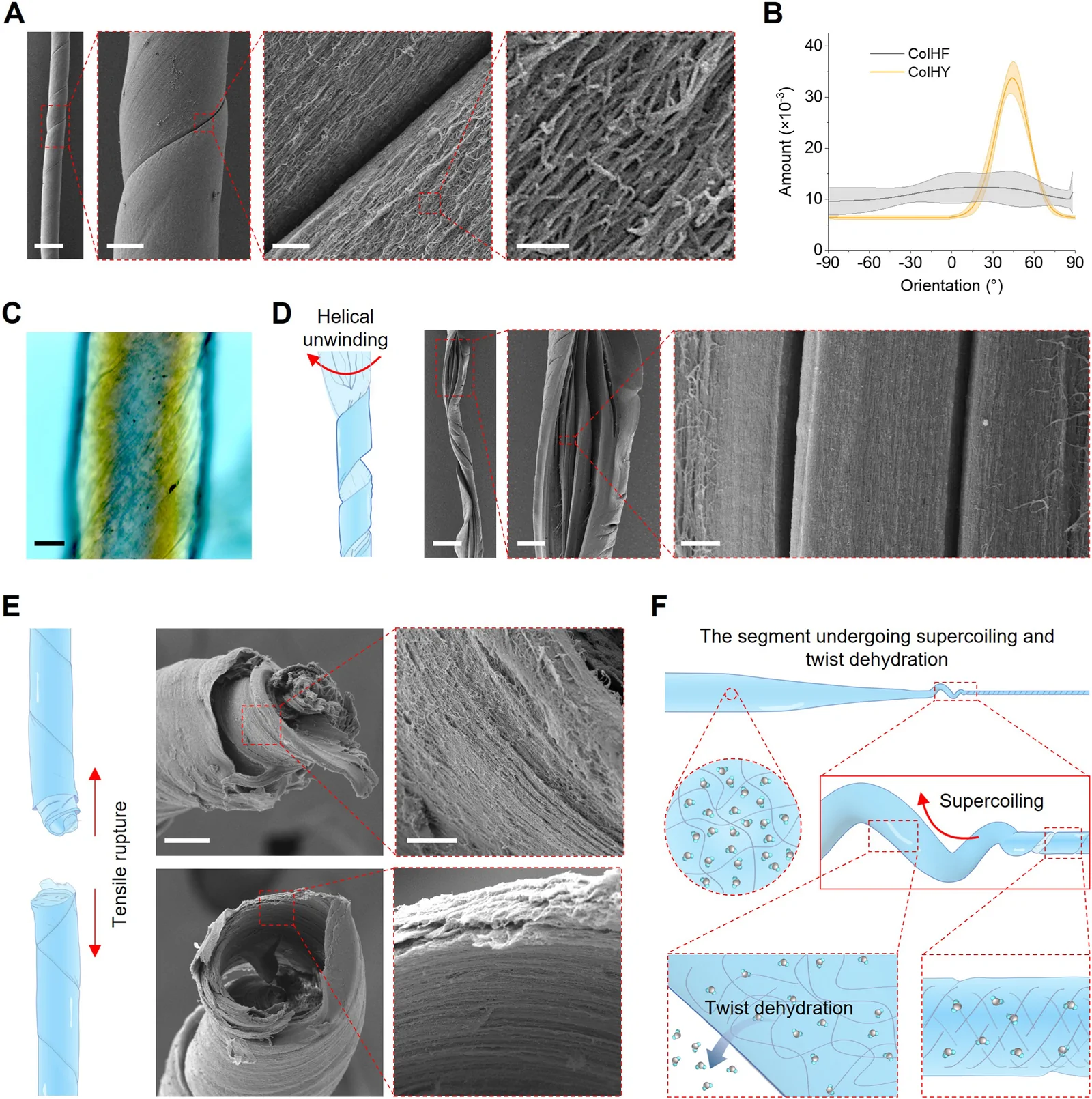
Twist-induced densification produces a hierarchical superhelical architecture with aligned collagen fibrils. (**A**) SEM images of the ColHY surface at increasing magnification, showing fibril alignment along the twisting direction. The surface helix angle $\theta_{s}$ (a key parameter in the scaling analysis) was extracted from images of this type. Scale bars: 200 μm, 50 μm, 2 μm, 500 nm (left to right). (**B**) Fibril orientation distributions for ColHFs (isotropic) and ColHYs (strongly aligned), quantified from SEM images. (**C**) Polarized light microscopy of a hydrated ColHY, confirming that the structural anisotropy observed in SEM is intrinsic to the wet material and not an artifact of lyophilization. Scale bar: 50 μm. (**D**) SEM images of a helically unwound ColHY, revealing internal bundle-like wrinkles composed of hierarchical sub-bundles oriented helically around the yarn axis. Scale bars: 200 μm, 50 μm, 2 μm (left to right). (**E**) SEM images of the tensile fracture surface, showing the multi-scale superhelical configuration in cross-section: collagen fiber bundles spiral around the central yarn axis. Scale bars: 50 μm (left), 10 μm (right). (**F**) Schematic of the staged structural remodeling during twisting: macroscopic helix formation, progressive radial compaction with water expulsion, and consolidation into the final superhelical yarn architecture.

SEM of lyophilized specimens revealed that twisting fundamentally reorganizes the collagen fibril network. Whereas ColHFs display a disordered, isotropic fibrillar architecture (Fig. S3), ColHYs exhibit highly aligned fibrils oriented along the twisting direction (Fig. 2A). Quantitative image analysis confirmed this pronounced alignment (Fig. 2B), and polarized light microscopy of hydrated ColHYs showed strong, uniform birefringence along the longitudinal axis, indicating that structural anisotropy is intrinsic to the wet material and not an artifact of lyophilization (Fig. 2C). SEM imaging of helically unwound ColHYs revealed further hierarchical organization: the internal surfaces displayed bundle-like wrinkles composed of sub-bundles, all oriented helically around the central yarn axis (Fig. 2D). Cross-sectional images of tensile fracture surfaces confirmed a multi-scale superhelical configuration in which collagen fiber bundles spiral around the yarn core (Fig. 2E).

These observations point to a staged formation mechanism (Fig. 2F). During the initial phase of twisting, the ColHFs develop a macroscopic helical configuration along their longitudinal axis. Continued twisting progressively reduces the helix radius, expelling interstitial water and driving radial compaction. This dehydration consolidates the collagen fibrils into the densified superhelical architecture of the final yarn. The result is a hierarchical structure with three features that prove central to the mechanical analysis developed below: (*i*) a high solid fraction set by the geometric compaction ratio ($R_{0}/R$)^2^; (*ii*) enhanced fibril alignment arising from a random-to-oriented transition; and (*iii*) a superhelical fiber architecture characterized by a helix angle θ that increases from zero at the yarn center to a maximum value $\theta_{s}$ at the surface. This architecture featuring aligned, densified collagen fibrils spiraling around a central axis is analogous structurally to the hierarchical organization of native tendon [44,45], suggesting that the mechanical behavior of ColHYs may similarly reflect both the material properties of the constituent network and the geometric properties of the superhelical arrangement.

### 3.2 MPa-scale mechanics from kPa-scale hydrogels

**Fig. 3:**
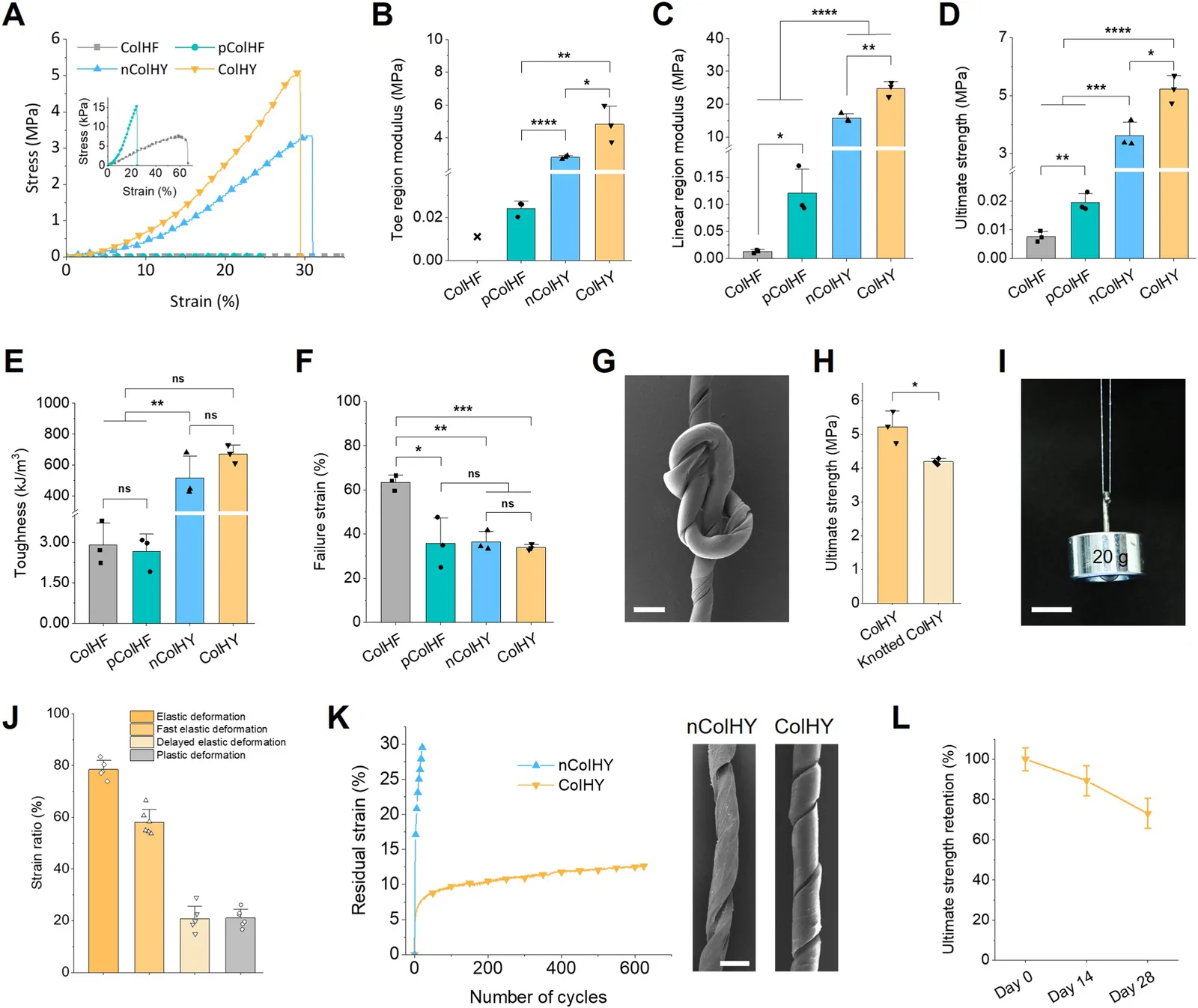
Twist-induced densification enhances tensile modulus, strength, and toughness by two to three orders of magnitude and produces tendon-like nonlinear strain-stiffening. (**A**) Tensile stress–strain curves for ColHFs, pColHFs, nColHYs, and ColHYs. The ColHY curve exhibits distinct toe and linear regions characteristic of native tendon; the toe-to-linear transition strain reflects progressive straightening of the superhelical architecture. (**B-F**) Mechanical properties extracted from the stress–strain curves: (B) toe-region modulus, (C) linear-region modulus, (D) ultimate tensile strength, (E) toughness, and (F) failure strain. “×” in (B) indicates that the ColHF toe-region modulus was below the measurement threshold. (**G**) SEM image of a knotted ColHY. Scale bar: 200 μm. (**H**) Knot strength, showing 81% retention of ultimate tensile strength. (**I**) A single ColHY (∼200 μm diameter) supporting a 20 g weight. Scale bar: 10 mm. (**J**) Elastic and plastic deformation fractions derived from constant-elongation elastic-recovery tests. (**K**) Residual strain during cyclic tensile loading. nColHYs failed within 20 cycles, whereas ColHYs withstood hundreds of cycles and exhibited a two-phase profile characterized by rapid initial accumulation followed by a near-stable plateau. Representative SEM images acquired after 10 loading cycles show structural deterioration in nColHYs and preservation of the yarn architecture in ColHYs. (**L**) Ultimate tensile strength retention of ColHYs after 0, 14, and 28 days of incubation in complete culture medium, expressed relative to the mean day-0 value. Scale bar: 200 μm. Statistical significance: *p < 0.05, **p < 0.01, ***p < 0.001, ****p < 0.0001; ns, not significant (Student's t-test). Data are presented as mean ± SD (n = 3 independent fabrication batches).

Quasi-static tensile tests of K_2_PtCl_4_-treated samples revealed that twist-induced densification markedly transformed the mechanical response of collagen hydrogels, increasing their modulus, strength, and toughness by two to three orders of magnitude (Fig. 3). The ColHFs and pColHFs exhibited tensile stresses confined to the kPa range, consistent with the dilute, isotropic networks observed. In contrast, both nColHY and ColHY samples reached the MPa scale (Fig. 3A and B; Fig. S4). The linear-region tensile modulus increased from 13.3 ± 3.2 kPa in ColHFs to 24.7 ± 2.3 MPa in ColHYs, a factor of >1800 (Fig. 3C). Ultimate tensile strength rose comparably, from 7.6 ± 1.9 kPa to 5.2 ± 0.5 MPa (a 690-fold increase; Fig. 3D), and toughness increased from 2.9 ± 0.8 to 671 ± 58 kJ/m^3^ (a 230-fold increase; Fig. 3E). These enhancements place ColHYs well above previously reported collagen hydrogel constructs, including plastically compressed collagen [26] and gel-aspirated ejected collagen [27], with their modulus, strength, and toughness approaching the lower range reported for native tendons.

The stress-strain response of ColHYs displayed pronounced nonlinear strain-stiffening, with distinct toe and linear regions characteristic of native tendon and ligament (Fig. 3A) [10,11]. The toe-region modulus of ColHYs was 4.8 ± 1.1 MPa, compared to 24.1 ± 3.4 kPa for pColHFs; the toe-region modulus of the dilute ColHFs was too low to measure reliably (Fig. 3B). This nonlinear behavior, which was absent in the isotropic as-fabricated hydrogels and emergent only after twisting, is a hallmark of the superhelical architecture identified in Section 3.1. In native tendon, the toe region arises from progressive straightening of crimped collagen fibrils under load [44,45]; the analogous structural motif in ColHYs is the superhelical fiber path, which must straighten before the constituent fibrils bear load directly. The strain at which the toe-to-linear transition occurs is therefore a geometric signature of the helix angle, a connection we develop quantitatively below. Failure strain was reduced in ColHYs relative to ColHFs (from 61 ± 3% to 35 ± 3%; Fig. 3F), a consequence of the constrained, densified network. However, this reduction was recovered by fabricating helical yarn variants, which exhibit extensibility exceeding 500% (Fig. S5).

The contributions of crosslinking and geometric transformation can be disentangled by comparing the intermediate processing states. The first crosslinking step (pColHF vs. ColHF) increased the linear-region modulus by approximately 9-fold, from 13.3 kPa to 0.121 MPa, while the second crosslinking step (ColHY vs. nColHY) stabilized the remodeled architecture with a further ∼1.6-fold increase. The dominant contribution, namely the ∼130-fold enhancement of the linear-region modulus from pColHF (0.121 MPa) to nColHY (15.8 MPa), arose entirely from the twist-induced densification step, with crosslinking held approximately constant. This step simultaneously compacts the network (increasing solid fraction by ∼80-fold), reorganizes the fibril orientation from isotropic to preferentially aligned, and imposes superhelical fiber geometry. How these three geometric factors quantitatively account for the observed ∼130-fold enhancement is addressed in the scaling analysis that follows.

Beyond quasi-static tensile performance, ColHYs exhibited the robustness required for textile processing and cyclic loading environments. Knotted ColHYs retained 81% of their ultimate tensile strength (4.2 ± 0.1 MPa vs. 5.2 ± 0.5 MPa; Fig. 3G–I), demonstrating tolerance to the bending and torsional stresses imposed during weaving and braiding. Stress relaxation measurements showed that ColHYs retained 64 ± 1% of their initial stress after 1200 s at 20% strain, compared to 51 ± 2% for nColHYs, indicating that the secondary crosslinking substantially enhances elastic storage (Fig. S6). Deconvolution of elastic recovery at constant elongation revealed that ∼79% of total deformation was elastic (58% fast elastic and 21% delayed elastic), with only 21% plastic (Fig. 3J; Fig. S7). Under cyclic tensile loading, nColHYs failed within 20 cycles, whereas ColHYs endured hundreds of cycles with a characteristic two-phase residual strain profile: rapid initial strain accumulation followed by a stable equilibrium phase (Fig. 3K; Fig. S8). ColHYs also exhibited enhanced resistance to enzymatic degradation (Fig. S9). Moreover, after 28 days of incubation in complete culture medium, ColHYs retained ∼73% of their initial ultimate tensile strength while exhibiting increased extensibility relative to day 0 (Fig. 3L; Fig. S10). Taken together, the high knot-strength retention, elastic recovery, cyclic mechanical stability, and long-term strength retention establish that ColHYs possess the mechanical resilience necessary for both textile fabrication and sustained load-bearing function.

### 3.3 Compaction geometry and network scaling quantitatively predict the enhancement

To isolate the mechanical contribution of twist-induced densification from that of standard chemical crosslinking, we modeled the modulus enhancement from pColHF (crosslinked but untwisted) to nColHY (twisted but not yet subjected to the secondary crosslinking step). This comparison holds the crosslinking state approximately constant and attributes the enhancement entirely to the geometric transformation. The measured linear-region moduli give $E_{nColHY}/E_{pColHF}\approx$ 15.8 MPa/0.121 MPa ≈ 130 (Fig. 3C). We show below that this ratio can be predicted from first principles using three physical ingredients, with no fitted parameters: densification, fibril alignment, and superhelical geometry.

For the first factor, densification during twisting, we note that the fiber radius decreases from $R_{0}\approx 900$ μm to R≈100 μm while collagen mass is approximately conserved per unit reference length. Assuming negligible axial dimensional change during densification, the cross-sectional-average solid volume fraction therefore increased according to $\varphi /\varphi_{0}∼\;{\left(R_{0}/R\right)}^{2}$, corresponding to an approximately 81-fold increase. Twist-driven solvent expulsion may generate a transient radial density gradient during densification. Because twisting was continued until the yarn diameter stabilized and cross-sectional SEM images showed an overall compact structure, the above relation is used here as a cross-sectional-average estimate rather than a description of the local radial density distribution. The latter was not directly quantified in the present study. In the densified ColHY, fibrils are preferentially aligned along the yarn axis and the network is sufficiently dense that deformation is approximately affine; that is, each fibril element stretches in proportion to the macroscopic strain. Under these conditions, the axial modulus scales linearly with solid fraction through the rule of mixtures: $E∼E_{f}\varphi$, where $E_{f}$ is the effective fibril modulus [49]. Notably, for consistency, we compare linear-region moduli in both pColHF and nColHY, which correspond to a stretching-dominated, quasi-affine response. The densification contribution to the modulus enhancement is therefore:

(1) $$\Gamma_{densification}=\frac{\varphi }{\varphi_{0}}\;∼\;{\left(\frac{R_{0}}{R}\right)}^{2}\approx 81$$

This is a substantial difference, but clearly does not account for the observed stiffening. We next considered reorganization of the fibrils from an initially random configuration of pColHF into a preferentially aligned configuration (Fig. 2A and B). For uniaxial tension applied to such a network, only the component of each fibril's stiffness projected onto the loading axis contributes to the macroscopic modulus, with each element's contribution scaling as $\cos^{4}\;\theta$, where θ is the angle between the loading axis and the fibril axis [49]. The orientation average $\left\langle \cos^{4}\;\theta \right\rangle$ depends on the dimensionality of the random distribution, with ${\left\langle \cos^{4}\;\theta \right\rangle }_{3D}=$ 1/5 for a fully 3D random distribution [50]. The other extreme to study for the purposes of bounding the scaling relationship is a fully random planar distribution, for which ${\left\langle \cos^{4}\;\theta \right\rangle }_{2D}=$ 3/8. The reciprocal of these gives the modulus gain from redirecting fibril stiffness toward the loading axis:

(2) $$\Gamma_{alignment}=\frac{1}{\left\langle \cos^{4}\;\theta \right\rangle }\approx 2.7∼5$$

Finally, in the twisted yarn, the collagen fibrils are not aligned axially but rather spiral at a helical angle θ(r)=arctan(2πτr), where τ is the twist density and r is the radial position [51]. Fibrils oriented off-axis contribute less to the axial modulus, with each element's contribution scaling as $\cos^{4}\;\theta$ from classical fiber-composite theory. Averaging $\cos^{4}\;\theta$ over the yarn cross-section, weighted by the area element 2πrdr, yields an effective stiffness correction factor:

(3) $$\Gamma_{helix}=\frac{2}{R^{2}}\;\int_{0}^{R}r\;\cos^{4}\left(\arctan\left(2\pi \tau \;r\right)\right)dr$$

Using the identity $\cos^{4}\left(\arctan\left(x\right)\right)={\left(1+x^{2}\right)}^{-2}$, where x=r/ρ, in which 1/ρ=2πτ

(4) $$\Gamma_{helix}=\frac{\rho^{2}}{R^{2}}\;\int_{0}^{R/\rho }\frac{2x}{{\left(1+x^{2}\right)}^{2}}dx=\frac{1}{1+{\left(R/\rho \right)}^{2}\;}=\cos^{2}\theta_{s}\approx 0.4∼0.6$$

where $\theta_{s}$ (∼40°-50°, Fig. 2A and B) is the helix angle at the yarn surface, meaning that the area-averaged off-axis stiffness reduction $\Gamma_{helix}$ across the entire cross-section reduces to a single function of the surface helix angle alone.

Combining the three factors gives:

(5) $$\frac{E_{nColHY}}{E_{pColHF}}=\Gamma_{densification}\Gamma_{alignment}\Gamma_{helix}∼{\left(\frac{R_{0}}{R}\right)}^{2}\frac{\cos^{2}\theta_{s}}{\left\langle \cos^{4}\;\theta \right\rangle }$$

giving an enhancement of ∼90-240. The experimentally measured ∼130-fold enhancement lies within this parameter-free prediction range.

The complete ColHF-to-ColHY transformation includes two additional contributions from chemical crosslinking: a first crosslinking step (K_2_PtCl_4_ for 10 min) stiffens the dilute isotropic ColHF network by $\alpha_{1}\approx$ 9-fold to create pColHF, prior to twisting; and a second crosslinking step (K_2_PtCl_4_ for 60 min) that stiffens nColHY by $\alpha_{2}\approx$ 1.6-fold to create ColHY. The final stiffening is:

(6) $$\frac{E_{ColHY}}{E_{ColHF}}=\alpha_{1}{\left(\frac{R_{0}}{R}\right)}^{2}\frac{\cos^{2}\theta_{s}}{\left\langle \cos^{4}\;\theta \right\rangle }\alpha_{2}$$

The superhelical geometry also provides a parameter-free explanation for the nonlinear strain-stiffening documented in Section 3.2. In the toe region, applied strain is accommodated by progressive straightening of the helical fiber paths rather than by stretching of the collagen fibrils themselves. The geometric strain required to straighten a fiber at helix angle θ is ε=(1/cosθ)−1. Because θ increases from zero at the yarn center to $\theta_{s}$ at the surface, different radial layers straighten sequentially, producing the gradually stiffening response observed experimentally. The transition to the linear region where fibrils bear load directly occurs when the outermost fibers are fully straightened, at a predicted transition strain of:

(7) $$\varepsilon_{\text{linear}}=\frac{1}{\cos\;\theta_{s}}-1$$

For $\theta_{s}\approx$ 40°-50°, this gives $\varepsilon_{\text{linear}}\approx 0.31-0.56$, which overlaps with and extends beyond the measured failure-strain range. This suggests that the yarn may still be in the process of stiffening when failure occurred, and that macroscopic failure of the yarn can be triggered prematurely by the rupture of the fully extended core fibrils, while the outer helical sheath is still mechanically compliant. This mechanism is analogous to the structural origin of the toe region in native tendon, where crimped collagen fibrils straighten progressively before engaging in direct extension [44,45]. It further suggests that tuning this effect may enable still higher moduli to be reached.

Finally, the approximately 230-fold increase in toughness, from 2.9 to 671 kJ/m^3^, can be understood as a compound effect. Although the failure strain of ColHYs is reduced relative to ColHFs, the orders-of-magnitude increase in both moduli more than compensates. The substantial toe region arising from the helical architecture provides significant energy absorption prior to irreversible fibril deformation, contributing to both the high toughness and the fatigue resistance observed under cyclic loading (Fig. 3K).

Taken together, this analysis suggests that mechanical enhancement is driven by the compaction geometry and established network mechanics. Two process-controllable parameters, namely compaction ratio $R_{0}/R$ and twist density τ (which determines $\theta_{s}$), determine the modulus, the constitutive nonlinearity, and the toughness. This renders twist-induced densification a designable process: the mechanical properties of the resulting yarn can be rationally predicted and tuned from fabrication parameters. One practical consequence of this designability is that yarns can be engineered to meet the specific mechanical thresholds required for textile processing, as we demonstrate next.

### **3.4** *Textile-grade processability*

**Fig. 4:**
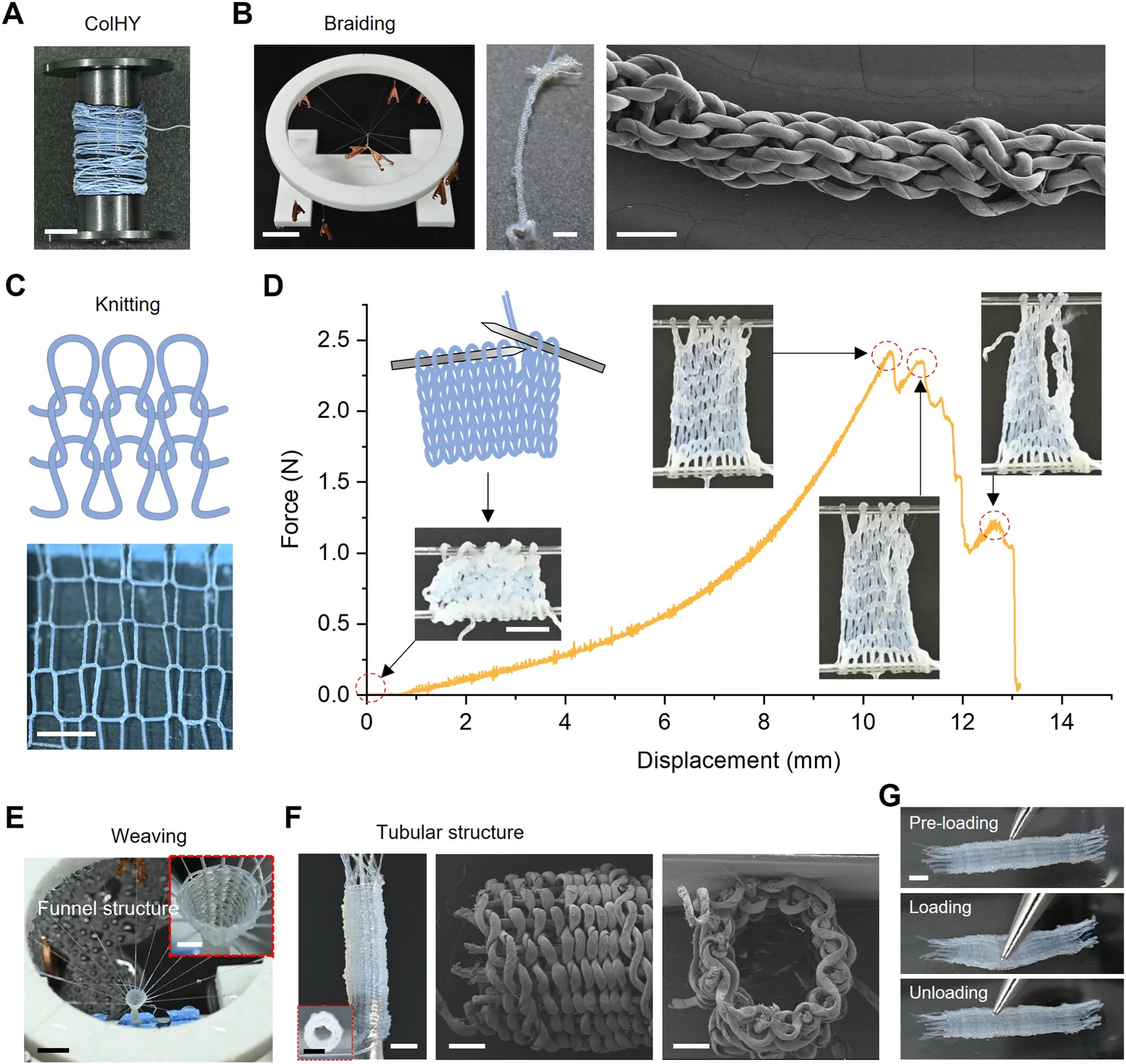
ColHYs possess textile-grade processability, enabling braided, knitted, and woven architectures spanning one to three dimensions. (**A**) Optical images of ColHYs as yarn-scale building blocks. Scale bar: 5 mm. (**B**) Braided structure at increasing magnification, demonstrating interlacing of multiple ColHY groups at controlled bias angles. Scale bars: 2 cm, 2 mm, 500 μm (left to right). (**C**) Planar knitted textile. Scale bar: 5 mm. (**D**) Force–displacement response of a knitted structure (ColHY diameter ∼400 μm; gauge length 6 mm; displacement rate 25 μm/s), with inset images at corresponding deformation states. The structure transmits force across interlocked yarns and continues to bear load after rupture of individual yarns, characteristic advantages of textile over monolithic architectures. Scale bar: 5 mm. See also Movies S3 and S4. (**E**) Funnel-shaped woven construct. Scale bars: 10 mm; 2 mm (inset). (**F**) Tubular woven construct at increasing magnification, showing the interlaced warp-weft architecture. Scale bars: 2 mm, 500 μm, 500 μm (left to right). (**G**) Compressive loading sequence of the tubular construct, demonstrating elastic recovery without luminal collapse, a property relevant to vascular and hollow-organ scaffold applications. Scale bar: 2 mm. See also Movie S5.

The combination of high tensile strength, knot retention, and fatigue resistance that arise as direct consequences of the compaction ratio and superhelical geometry enables ColHYs to serve as structural building blocks for textile fabrication (Fig. 4A). We demonstrate three canonical textile architectures of increasing dimensionality: braiding, knitting, and weaving.

Braided structures were formed by interlacing multiple ColHYs at controlled bias angles (Fig. 4B). Planar knitted textiles offered tunable porosity and extensibility (Fig. 4C and D). Tensile testing of a knitted structure (ColHY diameter ≈ 400 μm, gauge length 6 mm, displacement rate 25 μm/s) demonstrated load transfer across the interlocked textile architecture. The structure continued to sustain load even after rupture of individual yarns, a characteristic advantage of interlocked textile geometries over monolithic constructs (Fig. 4D; Movie S3). To enable quantitative mechanical characterization, a simplified knitted ColHY construct was further fabricated and tested (Fig. S11A; Movie S4). Under quasi-static stretching, the construct exhibited a linear-region modulus of 15.9 ± 4.9 MPa, a maximum nominal engineering stress of 3.4 ± 0.8 MPa, and a failure strain of 63 ± 5% (Fig. S11B). Suture-pullout testing yielded a maximum pullout force of 0.27 ± 0.04 N, corresponding to a suture-retention ratio of approximately 70% (Fig. S11C). In a representative cyclic tensile test, the simplified knitted construct withstood more than 250 loading cycles before failure (Fig. S11D). Together, these results demonstrate that textile assembly of ColHYs provides extensible and porous constructs capable of redistributing load through interlocked yarns, features that may be advantageous for load-bearing tissue-engineering applications.

Weaving was further used to generate 3D architectures, including funnel-shaped and tubular constructs (Fig. 4E and F). The tubular constructs exhibited durable mechanical resilience under compression, maintaining structural integrity and recovering elastically without luminal collapse (Fig. 4G; Movie S5), a property relevant to vascular and tubular organ engineering.

These demonstrations establish that ColHYs possess the full suite of mechanical properties required for textile processing, including tensile strength sufficient for weaving tension, flexibility adequate for braiding and knitting, and toughness to withstand repeated manipulation. Unlike synthetic polymer or electrospun scaffolds that achieve textile processability at the expense of biological permissiveness, ColHYs retain the native nanofibrillar architecture and protein bioactivity of the collagen matrix throughout the fabrication process [[52], [53], [54], [55]].

### 3.5 Preserved bioactivity and mechanical signal transmission

The twist-induced densification process subjects collagen hydrogels to extreme radial compaction and an 80-fold reduction in cross-sectional area, raising the question of whether the native bioactivity of the collagen matrix survives this transformation. To test this, fibroblasts were encapsulated within ColHFs prior to twisting, such that cells were present throughout the densification and crosslinking processes.

**Fig. 5:**
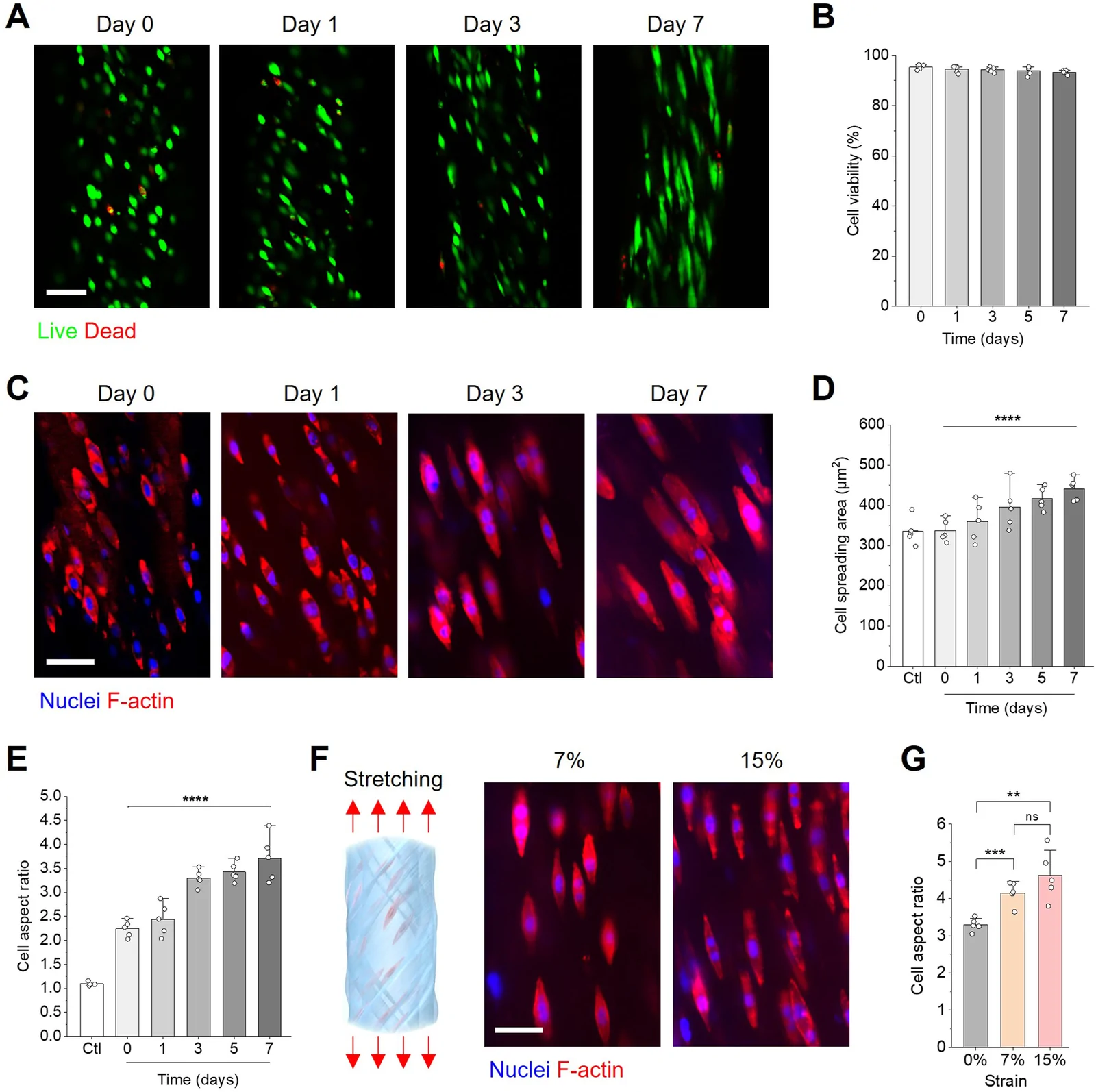
Application of ColHYs in 3D cell culture. (**A**) Representative live/dead staining images of cells encapsulated and cultured within ColHYs (green: live cells; red: dead cells). Scale bar, 100 μm. (**B**) Quantitative analysis of cell viability as a function of culture time. (**C**) Representative cytoskeleton staining images of cells cultured within ColHYs (red: F-actin; blue: nuclei). Scale bar, 50 μm. (**D**) Quantitative analysis of cell spreading area. (**E**) Quantitative analysis of cell aspect ratio. ****indicates statistical significance at *p* < 0.0001 (Spearman's rank correlation test). (**F**) Representative cytoskeleton staining image of cells within ColHYs after stretching to different strain levels, following 3 days of culture (red: F-actin; blue: nuclei). Scale bar, 50 μm. (**G**) Quantitative analysis of cell aspect ratio after stretching. Statistical significance: **p < 0.01, ***p < 0.001; ns, not significant (Student's t-test). Data are presented as mean ± SD (n = 5 independent cell-seeding and culture experiments).

Live/dead staining revealed that encapsulated cells maintained high viability (>90%) over seven days of culture (Fig. 5A and B). This high viability was also observed in stem cell encapsulation cultures (Fig. S12). This tolerance to the fabrication process likely reflects the plastic remodeling character of the collagen network during twisting: although the bulk geometry changes dramatically, local fibril rearrangements accommodate the compaction without transmitting crushing stresses to the cells. The small final diameter of the ColHYs (∼200 μm) may help limit diffusion distances for oxygen and nutrients within the construct [56].

The confined and anisotropic microenvironment of ColHYs was associated with pronounced changes in cell morphology. Cells that initially exhibited a near-spherical morphology within the relatively isotropic ColHFs (Fig. S13) became elongated along the longitudinal direction following twist-induced densification (Fig. 5C). Both cell spreading area and aspect ratio increased progressively over seven days (Fig. 5D and E), while 3D confocal reconstruction confirmed that cells were distributed throughout the yarn thickness and preferentially aligned along the yarn axis (Fig. S14). These observations suggest that the structural microenvironment generated by densification, including fibrillar anisotropy and spatial confinement, promoted cytoskeletal organization without additional exogenous biochemical or mechanical stimulation.

To determine whether ColHYs can also transmit externally applied mechanical loads to encapsulated cells, cell-laden yarns cultured for three days were subjected to tensile strains of 7% or 15% for 1 h. Stretched cells exhibited significantly enhanced elongation relative to unstrained controls, with cell aspect ratio increasing in a strain-dependent manner (Fig. 5F and G). This response confirmed that the densified ColHY matrix couples macroscopic mechanical loading to cellular-scale deformation, a prerequisite for mechanotransduction-based tissue maturation strategies in which cyclic loading is used to drive phenotypic development [[57], [58], [59]].

These results establish that twist-induced densification, despite its severity, preserves two essential biological functions of a collagen hydrogel: a microenvironment conducive to cell survival, and a structurally anisotropic matrix capable of both contact-guided alignment and mechanical signal transmission. The combination of designable, tissue-scale mechanics with retained biological functionality positions ColHYs as a platform in which mechanical and biological performance can be achieved simultaneously rather than traded off against each other.

This study has several limitations. First, the biological evaluation was limited to relatively short-term in vitro culture. Seven-day viability measurements cannot exclude delayed cytotoxicity, inflammatory responses, or other chronic effects associated with K_2_PtCl_4_, nor can they establish sustained cell survival and function within the densified yarns. 3D confocal reconstruction at day 7 showed cells distributed across the yarn cross-section without an obvious central cell-free region or outer-shell-restricted distribution; however, these observations do not exclude the development of diffusion limitations or a necrotic core during longer-term culture. K_2_PtCl_4_ was used here as a model crosslinker to stabilize the remodeled collagen network and is not intrinsic to the twist-densification process. The successful fabrication of genipin-crosslinked ColHYs, which retained high cell viability and robust mechanical properties (Fig. S15), supports the transferability of the strategy to an alternative crosslinking chemistry. However, these findings do not substitute for systematic long-term safety assessment. Longer-term viability, metabolic and functional analyses, together with in vivo evaluation, will therefore be required. Second, although the system was shown to work with primary rBMSCs, most cell experiments were performed using NIH/3T3 fibroblasts; using primary tendon fibroblasts/tenocytes in the future would be desirable. In addition, although cells became preferentially aligned within the densified yarns, the relative contributions of fibrillar anisotropy and spatial confinement to this morphological response were not independently resolved. Finally, the textile constructs demonstrated here were primarily proof-of-concept architectures. Translation toward tissue-engineering applications will require anatomically relevant textile designs, evaluation under application-specific mechanical loading, and in vivo assessment of integration, remodeling, degradation, and functional regeneration.

## 4 Conclusion

Results demonstrate that twist-induced densification transforms dilute collagen hydrogels into superhelical yarns with tensile moduli, strengths, and toughness values enhanced by two to three orders of magnitude, bringing the mechanical properties of reconstituted collagen toward the lower range reported for native tendon. The enhancement is predictable quantitatively: by isolating the geometric transformation from chemical crosslinking, we show that the dominant ∼130-fold contribution of twist-induced densification is accounted for quantitatively by three factors from fiber-composite mechanics, namely network densification, the random-to-aligned fibril transition, and the helical fiber geometry. The surface helix angle that enters the modulus prediction independently predicts the constitutive nonlinearity. This mechanistic understanding renders the process rationally tunable, enabling selection of yarn mechanics from fabrication parameters.

Two practical consequences follow. First, the rationally tunable mechanics are sufficient for textile-grade processing using yarns composed entirely of self-assembled collagen, including braiding, knitting, and weaving into architectures spanning one to three dimensions. Second, the bioactivity of the collagen matrix survives the densification process, supporting 3D cell encapsulation with high viability, alignment, and strain-responsive mechanical coupling.

Looking forward, the scaling framework developed here should extend beyond collagen to other reconstituted protein hydrogels (fibrin, silk fibroin, elastin) in which mechanical performance is governed by fibril density, orientation, and architecture. The most immediate opportunities lie in combining automated textile fabrication with cell-laden ColHYs to produce living tissue constructs whose architecture and mechanics are prescribed by design, and in exploiting the strain-dependent mechanical coupling demonstrated here for cyclic-loading protocols that drive functional tissue maturation.

## CRediT authorship contribution statement

**Yang Xie:** Writing – original draft, Investigation, Formal analysis, Data curation. **Wenjie Wu:** Writing – original draft, Investigation, Formal analysis, Data curation. **Weiwei Zhang:** Investigation, Funding acquisition. **Xiangjun Peng:** Writing – review & editing, Methodology. **Tiancai Sun:** Methodology. **Yanling Liu:** Writing – review & editing, Methodology. **Zuoqi Zhang:** Writing – review & editing, Funding acquisition. **Elliot L. Elson:** Writing – review & editing. **Guy M. Genin:** Writing – review & editing, Validation, Methodology. **Guoyou Huang:** Writing – review & editing, Validation, Supervision, Methodology, Funding acquisition, Conceptualization.

## Data availability statement

Raw and processed data are available from the corresponding author upon reasonable request.

## Ethics approval and consent to participate

This study did not involve human participants, human data, or human tissue, nor did it involve animals. All cell lines used were commercially obtained and authenticated. Therefore, no ethical approval or consent to participate was required for this study.

## Declaration of competing interest

The authors declare the following financial interests/personal relationships which may be considered as potential competing interests: Guoyou Huang, Yang Xie, Wenjie Wu, Weiwei Zhang have patent pending to Wuhan University. The other authors declare that they have no known competing financial interests or personal relationships that could have appeared to influence the work reported in this paper.
