# Supplementary material for: A designable twist-densification route to bioactive collagen hydrogel yarns approaching tendon-like mechanics
Source: Bioact Mater. 2026 Sep 17;68:835–47. doi: 10.1016/j.bioactmat.2026.09.010 (PMC13599669; doi:10.1016/j.bioactmat.2026.09.010)
Supplement: Supplementary file 1 [file mmc1.docx]

**Fig. S1.** Fabrication of collagen hydrogel yarns (ColHYs) with controlled diameters. (**A**) ColHFs fabricated using silicone tubes with inner diameters of 3 mm (left) and 4 mm (right). Scale bar: 1 cm. (**B**) Representative optical images of ColHYs derived from the ColHFs shown in (A). Scale bar: 500 μm. (**C**) Quantitative correlation between the final ColHY diameter and the initial ColHF diameter. Data are presented as mean ± SD (n = 5 independent fabrication batches).

**Fig. S2.** Mass fraction analysis of ColHYs with controlled diameters. ColHY1, ColHY2, and ColHY3 are designated as ColHYs fabricated from ColHFs extruded through silicone tubes with inner diameters of 2 mm, 3 mm, and 4 mm, respectively. Data are presented as mean ± SD (n = 5 independent fabrication batches).

**Fig. S3.** Structural characterization of ColHFs. Scale bar: 1 μm.

**Fig. S4.** Representative load–displacement curves for ColHFs, pColHFs, nColHYs, and ColHYs.

**Fig. S5.** Fabrication and characterization of helical ColHYs. (**A**) Representative optical image of a helical ColHY. Scale bar: 5 mm. (**B**) Representative SEM image of a helical ColHY. Scale bar: 500 μm. (**C**) Representative tensile stress-strain curve of a helical ColHY. Insets show corresponding images of the helical ColHY at 0%, 100%, and 500% tensile strain.

**Fig. S6.** Stress-relaxation behavior of nColHYs and ColHYs at 20% strain. (**A**) Representative normalized stress–time responses. (**B**) Residual stress ratio after 1200 s. **** indicates statistical significance at *p* < 0.0001 (Student’s t-test). Data are presented as mean ± SD (n = 5 independent fabrication batches).

**Fig. S7.** Elastic recovery test at constant elongation. Representative stress-strain curve of ColHYs under elastic recovery test at constant elongation, with *ɛ*_0_, *ɛ*_1_, and *ɛ*_2_ indicated for calculating elastic and plastic deformation ratios. The corresponding quantitative deformation fractions are presented in Fig. 3J.

**Fig. S8.** Representative cyclic normalized stress-strain curves comparing the performance of a nColHY and a ColHY during a tensile fatigue test.

**Fig. S9.** Degradation tests. Degradation was characterized by measuring sample diameters during incubation in collagenase solution. For each material condition, five independent experiments were performed, each with two technical replicates; the two measurements were averaged to obtain a single independent value (n = 5).

**Fig. S10.** Mechanical evolution of ColHYs during 28 days of incubation in complete culture medium. Representative engineering stress–strain curves of ColHYs are shown at days 0, 14, and 28. Quantitative analysis of ultimate tensile strength retention is presented in Fig. 3L.

**Fig. S11.** Quantitative mechanical characterization of a simplified knitted ColHY construct. (**A**) Schematic illustration of the simplified knitted ColHY construct. (**B**) Quasi-static tensile stress–strain responses of three independently fabricated simplified knitted constructs (n = 3). Engineering stress was calculated using the sum of the initial cross-sectional areas of the three ColHY yarns spanning the load-bearing cross-section. (**C**) Force–displacement curves obtained during suture-pullout testing of three independently fabricated constructs (n = 3). (**D**) Representative cyclic tensile response of a simplified knitted ColHY construct tested using the same normalized loading protocol as that used for individual ColHYs. The representative construct withstood more than 250 loading cycles before failure. The arrow indicates increasing cycle number.

**Fig. S12.** Viability of Sprague–Dawley rat bone marrow-derived mesenchymal stem cells (rBMSCs; passages 3–6) encapsulated in ColHYs. (**A**) Representative live/dead fluorescence images at days 0, 1, 3, and 7. Scale bar: 100 μm. (**B**) Quantitative analysis of cell viability from day 0 to day 7. Five independent cell-seeding and culture experiments were performed (n = 5). Data are presented as mean ± SD.

**Fig. S13.** Cell morphology just encapsulated in ColHFs. Scale bar: 50 μm.

**Fig. S14.** 3D confocal reconstruction of cellular distribution within ColHYs. (**A**) Representative 3D confocal reconstruction image showing cellular distribution after 7 days of culture within a ColHY. (**B**) Top-down (XY plane) and cross-sectional (XZ/YZ plane) views of the 3D reconstruction shown in (A). Scale bar: 100 μm.

**Fig. S15.** Cytocompatibility and mechanical properties of genipin-crosslinked ColHYs. (**A**) Representative live/dead fluorescence images of NIH/3T3 fibroblasts encapsulated in genipin-crosslinked ColHYs during 7 days of culture. Scale bar: 100 μm. (**B**) Quantitative cell viability over 7 days. Cell viability remained above 95% throughout the observation period (n = 5 independent cell-seeding and culture experiments). (**C**) Representative engineering stress–strain curve of genipin-crosslinked ColHYs. Genipin-crosslinked ColHYs exhibited a toe-region modulus of 10.3 ± 0.4 MPa, a linear-region modulus of 49.3 ± 3.0 MPa, an ultimate tensile strength of 7.2 ± 0.4 MPa, a failure strain of 25 ± 1%, and a toughness of 689 ± 28 kJ/m³ (n = 3 independent fabrication batches). Data are presented as mean ± SD.

**Movie S1.** Continuous twisting of collagen hydrogel fiber to form yarn.

**Movie S2.** Quasi-static tensile testing of an individual ColHY.

**Movie S3.** Tensile deformation of the knitted ColHY structure shown in Fig. 4D.

**Movie S4.** Quasi-static tensile testing of a simplified knitted ColHY construct.

**Movie S5.** Compressive deformation and elastic recovery of a tubular ColHY structure.
